# Effect of Different Surface Microstructures in the Thermally Induced Self-Propulsion Phenomenon

**DOI:** 10.3390/mi13060871

**Published:** 2022-05-31

**Authors:** Clint John Cortes Otic, Shigeru Yonemura

**Affiliations:** 1 Department of Finemechanics, Graduate School of Engineering, Tohoku University, 6-6 Aramaki Aza Aoba, Aoba-ku, Sendai 980-8579, Miyagi, Japan; 2 Department of Mechanical Engineering, College of Engineering, Chubu University, 1200 Matsumoto-cho, Kasugai 487-8501, Aichi, Japan

**Keywords:** micro/nano-scale flows, molecular gas dynamics, direct simulation Monte Carlo (DSMC) method, computational fluid dynamics, rarefied gas

## Abstract

In micro/nano-scale systems where the characteristic length is in the order of or less than the mean free path for gas molecules, an object placed close to a heated substrate with a surface microstructure receives a propulsive force. In addition to the induced forces on the boundaries, thermally driven flows can also be induced in such conditions. As the force exerted on the object is caused by momentum brought by gas molecules impinging on and reflected at the surface of the object, reproducing molecular gas flows around the object is required to investigate the force on it. Using the direct simulation Monte Carlo (DSMC) method to resolve the flow, we found that by modifying the conventional ratchet-shaped microstructure into different configurations, a stronger propulsive force can be achieved. Specifically, the tip angle of the microstructure is an important parameter in optimizing the induced force. The increase in the propulsive force induced by the different microstructures was also found to depend on the Knudsen number, i.e., the ratio of the mean free path to the characteristic length and the temperature difference between the heated microstructure and the colder object. Furthermore, we explained how this force is formed and why this force is enhanced by the decreasing tip angle, considering the momentum brought onto the bottom surface of the object by incident molecules.

## 1. Introduction

It is well known that non-uniform temperature fields induce convection in a continuum gas surrounding us. Here, convection is induced due to the combined effect of non-uniform density and gravity, i.e., buoyancy. However, if there is no external force such as gravity, convection will not occur because buoyancy will not appear since it is driven by gravity. Contrary to this, non-uniform temperature fields in a rarefied gas, such as that in micro/nano-scale flow or low-pressure conditions, can cause a steady flow even without external forces. This phenomenon appears in conditions where the Knudsen number, defined as the ratio of the mean free path for gas molecules to the characteristic length of the flow, is not vanishingly small [1]. Such flows are called thermally driven flows since they are induced by non-uniform temperature distributions. These include the thermal creep flow, thermal stress slip flow, nonlinear thermal stress flow, and thermal edge flow [2]. Thermally driven flows are the key mechanism in the operation of Knudsen pumps that provide a steady gas flow rate without using any moving parts [3]. The very simple structures of several micro-scale configurations of the Knudsen pump [4,5] make it attractive for research. The physics of thermally driven flows has been a subject of several analyses, from lid-driven cavity cases [6,7] to periodically patterned channel cases [8]. Furthermore, in addition to thermally driven flows, temperature distributions in rarefied gas can also generate macroscopic forces. Specifically, the profile of the temperature distribution in the gas can result in a force on the solid boundaries and a net gas flow [9]. These macroscopic forces can be in the form of radiometric forces [10] and thermophoretic forces [11], among other forces, which are collectively called Knudsen forces [12,13]. A significant number of studies on Knudsen forces have focused mainly on its normal component due to its potential applications in actuation devices [14,15]. However, several studies on a channel bounded by ratchet structured surfaces with different temperatures and whose characteristic length is in the order of or less than the mean free path for gas molecules have shown that the Knudsen force generated on the solid boundaries has not only a normal component but also a tangential component [16,17,18]. It was even suggested that the tangential component of the Knudsen force may have contributed to the self-propulsion of a Leidenfrost object placed on a heated ratchet surface [17,19]. The results from these previous studies [16,17,18,19] have suggested that if an object is placed close to a substrate with a surface microstructure, a force will be induced on the object. This force can set the object in motion without the use of moving parts, i.e., self-propulsion. As this force is acting on the surface of the object tangentially to it, and this force only appears in not so low Knudsen numbers, this propulsive force is referred to in this study as the tangential Knudsen force or the tangential Knudsen stress in the unit area. Moreover, it has been pointed out intuitively that the asymmetric nature of the ratchet structure is the origin of the propulsive force [16]. Therefore, by modifying the surface structure into different asymmetric configurations, the propulsive force can be controlled and optimized. In this paper, we present new configurations with asymmetric geometries as surface microstructures that are modifications to the conventionally used ratchet configuration. The results of this study will be useful in designing systems that utilize this self-propulsion.

## 2. Methodology

### 2.1. Configuration of the Surface Microstructures

**Figure 1:**
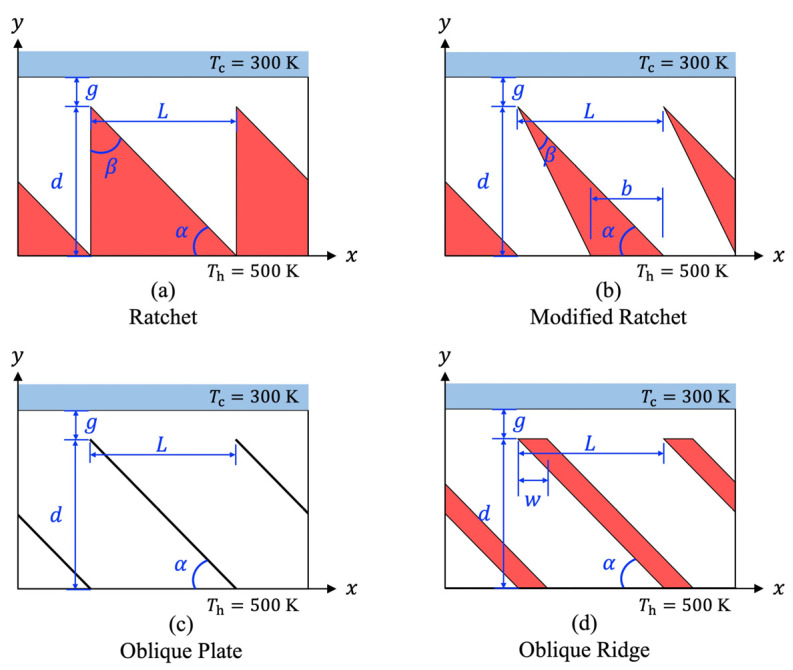
Schematics of the substrate with different surface microstructures.

We considered an infinitely wide object of cold temperature $T_{c}=300$ K placed close to an infinitely wide substrate of hot temperature $T_{h}=500$ K, which has a surface structure repeated periodically in the x -direction, as shown in Figure 1. The bottom surface of the object is perfectly flat and defines the upper boundary of the channel. On the other hand, the substrate with the periodically repeated surface structure of periodic length L and a depth d defines the lower boundary of the channel. The space between the bottom surface of the object and the substrate creates a channel that is filled with air. The minimum gap g between the bottom surface of the object and the tip of the surface structure of the substrate was set at g=0.2d .

The four configurations shown in Figure 1 are considered here as the surface structures. The first surface structure is called here as “ratchet” shown in Figure 1a, is defined by the periodic length L , an oblique wall with an inclination angle α, a vertical wall with a depth d given by d=Ltanα , and a tip angle β given by $\beta =\left(\pi /2\right)-\alpha$ . The second surface structure is called here as “modified ratchet” with the tip angle set $\beta <\left(\pi /2\right)-\alpha$ without changing the inclination angle α , as shown in Figure 1b. Note that this modification causes the vertical side of the ratchet to be adjusted into an inclined orientation. Let us call this the modified side and the other oblique wall, the oblique side, respectively. The third surface structure is called here as “oblique plate,” which is the limiting case of the modified ratchet, i.e., β=0 , as shown in Figure 1c. In this case, the modified side coincides with the oblique side. The fourth surface microstructure is called here as “oblique ridge,” which is a modification of the oblique plate in which a thickness w is added, as shown in Figure 1d. In this study, an aspect ratio of unity, i.e., d=L , is considered as the reference such that the inclination angle is α=π/4 . Furthermore, a base length b=0.5L is considered as the reference for the modified ratchet structure such that its tip angle is $\beta =\left(\pi /4\right)-\tan^{-1}0.5$ , while the width w=0.2L is used for the oblique ridge.

### 2.2. Direct Simulation Monte Carlo (DSMC) Method and Parameters

In this paper, we focused on flows outside the continuum flow regime, and therefore, the Navier–Stokes equation is not applicable. We used the direct simulation Monte Carlo (DSMC) method [20,21] to reproduce such flows. The DSMC method has already been used in several studies to simulate thermally driven flows in microchannels involving ratchet-like periodic structures [16,17,18]. This method is a particle-based algorithm in which real molecules are represented by a relatively small number of sample molecules that approximate the velocity distribution function of the real molecules, and the motions of sample molecules are simulated and followed. Although realistic air consists of many kinds of molecules, we represented the air molecules with an imaginary hard-sphere molecule [22] of mass $m=4.80967\times 10^{-26}$ kg and a diameter $d_{m}=0.356$ nm. The diameter $d_{m}$ was evaluated from a reference viscosity and using the reference temperature $T_{\mathrm{ref}}=\left(T_{h}+T_{c}\right)/2=400$ K [23,24]. For simplicity, we assumed that the diameter of hard-sphere molecules does not depend on the local temperature of the gas. Thus, in the present study, we discuss the Knudsen force not in realistic air but in the ideal case of a gas consisting of hard-sphere molecules with constant diameter. That is, the discussion in this study will not be strictly accurate for a realistic air. However, thanks to the usage of molecules of the constant diameter, which is not unique to air, the results obtained in the present simulations can be applied to other gases without being limited to air in the case wherein the Knudsen number is the same.

The reference mean free path $\lambda_{\mathrm{ref}}$ and the reference mean free time $\tau_{\mathrm{ref}}$ for molecules with a diameter $d_{m}=0.356$ nm under the condition of the reference atmosphere set at $p_{\mathrm{ref}}=101325$ Pa, and the reference temperature $T_{\mathrm{ref}}=400$ K, are $\lambda_{\mathrm{ref}}=96.7969$ nm and $\tau_{\mathrm{ref}}=1.790101\times 10^{-10}$ s, respectively. These are obtained by using the relations $\lambda =1/\sqrt{2}n\pi d_{m}^{2}$ and $p=nk_{B}T$ , where n is the number density and $k_{B}=1.380649\times 10^{-23}$ J/K is the Boltzmann constant. Note that $\tau_{\mathrm{ref}}$ is the expected mean free time, which corresponds to the mean free path $\lambda_{\mathrm{ref}}$ and is given by $\tau_{\mathrm{ref}}=\lambda_{\mathrm{ref}}/{\bar{C}}_{\mathrm{ref}}$ , where ${\bar{C}}_{\mathrm{ref}}=\sqrt{8k_{B}T_{\mathrm{ref}}/\pi m}$ is the mean molecular speed.

**Figure 2:**
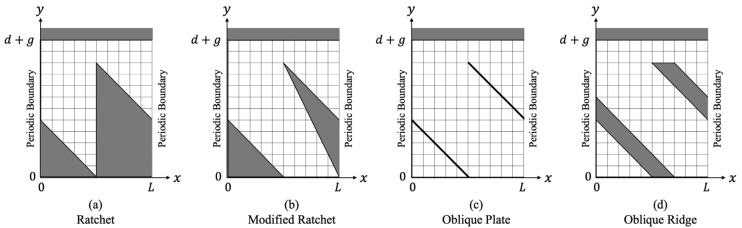
Computational domain used in each microstructure.

The gap distance g was used as the characteristic length to define the Knudsen number Kn , as $\mathrm{Kn}=\lambda_{\mathrm{ref}}/g$ . In this study, we considered different Knudsen numbers by keeping the gas mean free path $\lambda_{\mathrm{ref}}$ constant, i.e., the reference gas pressure $p_{\mathrm{ref}}$ is kept constant while adjusting the gap g as $g=\lambda_{\mathrm{ref}}/\mathrm{Kn}$ . For example, with the $\lambda_{\mathrm{ref}}$ given above, in the case of Kn=0.1 , the gap is g=967.9697 nm, and so on. In each of the surface microstructures, the flow was investigated at different Knudsen numbers enumerated in Table 1. The computational domain used for the DSMC in each microstructure is the region within one periodic length L along the x axis, i.e., 0≤x≤L , and 0≤y≤d+g . Here, y=0 is set at the bottom of the microstructure, and x=0 is set at the center of the oblique side, except for the oblique ridge where x=0 is set at the center of the left side of the oblique ridge, as shown in Figure 2. In addition, the side boundaries of the computational domain are considered to be periodic in the +x -direction and −x -direction. A uniform grid is used such that the computational domain was divided into rectangular cells with cell size Δx=Δy . The chosen cell sizes at different Knudsen numbers are enumerated in Table 1 and is set sufficiently smaller than the mean free path, at a maximum of $\lambda_{\mathrm{ref}}/3$ , for all the Knudsen number considered in this study. Moreover, the number of sample molecules in each DSMC cell was initialized at 50 for the standard cell size of Δx×Δy .

The main essence of the DSMC method is the uncoupling of the intermolecular collision from the free molecular motion over a small time step Δt . The time step Δt must be set much smaller than the mean free time $\tau_{\mathrm{ref}}$ for intermolecular collisions [20]. In this study, the chosen time step is enumerated in Table 1. Notice that for Knudsen numbers less than unity, Kn≤1 , the time step is set at $\Delta t=0.08\tau_{\mathrm{ref}}$ , which corresponds to a representative molecular displacement in the x -direction or y -direction of $\left({\bar{C}}_{\mathrm{ref}}/2\right)\Delta t=0.04\lambda_{\mathrm{ref}}$ . This is sufficiently smaller than the cell size Δx or Δy , that molecules do not travel across more than one cell size within one time step. For Knudsen numbers greater than unity, Kn>1 , consider the case where we keep the time step at $\Delta t=0.08\tau_{\mathrm{ref}}$ , i.e., the corresponding representative molecular displacement is kept at $0.04\lambda_{\mathrm{ref}}$ , while the cell size is $0.05g=0.05\lambda_{\mathrm{ref}}/\mathrm{Kn}$ . Under such conditions, the cell size decreases with increasing Kn and will be smaller than the representative molecular displacement, i.e., molecules can travel across more than one cell size within one time step. In order to avoid this, we set the time step for Kn>1 at $\Delta t=0.08g/{\bar{C}}_{\mathrm{ref}}$ instead, such that the representative molecular displacement is $\left({\bar{C}}_{\mathrm{ref}}/2\right)\Delta t=0.04g$ , which is smaller than the cell size of 0.05g . By doing so, the condition that molecules do not travel across more than one cell size within one time step is satisfied even for high Knudsen numbers.

The free motions and intermolecular collisions of molecules were tracked, and the time evolution of the flow field was simulated. In the present simulations, intermolecular collisions were judged stochastically based on the collision probability for the pair of sample molecules in each cell by using the maximum collision number method [25,26].

To elucidate this method, let us consider a single cell containing N number of sample molecules whose velocities are $c_{1}$ , $c_{2},$ ⋯ , $c_{N}$ . The probability that a sample molecule s will collide with another sample molecule k within a single time step Δt , $P_{s,k}$ , is

(1) $$P_{s,k}=N^{-1}ng_{s,k}\sigma_{T}\Delta t$$

where $g_{s,k}=\left|c_{k}-c_{s}\right|$ is the relative speed between the sample molecules s and k , n is the number density of real molecules in the considered cell, and $\sigma_{T}$ is the total collision cross-section given by $\sigma_{T}=\pi d_{m}^{2}$ . Then, the maximum collision probability, $P_{\max}$ , is evaluated using the estimated maximum relative speed between all possible pairs, $g_{\max}$ , such that

(2) $$P_{\max}=N^{-1}ng_{\max}\sigma_{T}\Delta t$$

In the present simulations, initially, the maximum relative speed was estimated as $g_{\max}=5\sqrt{k_{B}T_{\mathrm{ref}}/m}$ . In the case when a $g_{s,k}$ larger than $g_{\max}$ appeared, the value of $g_{\max}$ was replaced by the larger one. 

In the maximum collision number method, we consider that each pair of molecules has a collision probability of $P_{\max}$ . However, this is not a real collision. We call this type of collision with the probability of $P_{\max}$ as a tentative collision, in other words, a candidate of real collision. As the number of possible pairs of sample molecules in the cell is $N\left(N-1\right)/2$ , the number of tentative collision pairs is given by

(3) $$P_{\max}=N^{-1}ng_{\max}\sigma_{T}\Delta t\;\;=\left(N-1\right)ng_{\max}\sigma_{T}\Delta t/2$$

Here, $N_{\max}$ is called the maximum collision number. Then, we choose $N_{\max}$ tentative collision pairs randomly out of all $N\left(N-1\right)/2$ possible pairs of sample molecules. This procedure is equivalent to the procedure in that we choose tentative collision pairs based on the probability of $P_{\max}$ . Then, we judge whether each of the selected tentative collision pairs, e.g., $\left(s,k\right)$ , results in a real collision pair or not, based on the probability of $P_{s,k}/P_{\max}$ . Thus, this procedure is equivalent to the procedure that whether each of $N\left(N-1\right)/2$ possible pairs, e.g., $\left(s,k\right)$ , results in a real collision pair or not is judged based on the probability $P_{s,k}$ since $P_{\max}\times \left(P_{s,k}/P_{\max}\right)=P_{s,k}$ . The latter procedure is straightforward, but its computational load is proportional to $N\left(N-1\right)/2$ , i.e., roughly $N^{2}$ . Using a large number of sample molecules is an expensive task for the latter procedure. On the other hand, the former procedure, i.e., the maximum collision number method, is two-tiered, and its computational load is proportional to $N_{\max}$ , i.e., $\left\{N\left(N-1\right)/2\right\}\times P_{\max}$ . As $P_{\max}$ is inversely proportional to N as shown in Equation (3), the computational load of the maximum collision number method is proportional to N . Due to this characteristic of the maximum collision number method, we can use a large number of sample molecules.

In addition, for molecule–wall collisions at the solid boundaries, impinging gas molecules were scattered using the diffuse reflection model [22]. Within an infinitesimally small time length of molecule-surface interaction, all the molecules impinging on the surface were diffusively emitted after the full adaptation to the temperature of the surface.

After the gas flow was judged to have reached the steady state, the molecular velocities and the number of sample molecules started to be sampled at each DSMC cell, and the impulses given on the bottom surface of the upper object by colliding molecules started to be accumulated. The number density n , flow velocity v , and temperature T in each cell $\left(i,j\right)$ at the end of $M_{\mathrm{samp}}$ samplings are

(4) $$n_{i,j}=\frac{W_{f}}{V_{\mathrm{cell}}\left(i,j\right)}\left[\frac{N_{\mathrm{tot}}\left(i,j\right)}{M_{\mathrm{samp}}}\right]$$

(5) $$v_{i,j}=\frac{1}{N_{\mathrm{tot}}\left(i,j\right)}\overset{M_{\mathrm{samp}}}{\underset{l=1}{\sum }}\overset{N^{\left(l\right)}\left(i,j\right)}{\underset{q=1}{\sum }}c_{q}$$

(6) $$T_{i,j}=\frac{m}{3k_{B}}\left[\left(\frac{1}{N_{\mathrm{tot}}\left(i,j\right)}\overset{M_{\mathrm{samp}}}{\underset{l=1}{\sum }}\overset{N^{\left(l\right)}\left(i,j\right)}{\underset{q=1}{\sum }}c_{q}^{2}\right)-v_{i,j}^{2}\right]$$

Here, $V_{\mathrm{cell}}\left(i,j\right)$ is the volume of the cell $\left(i,j\right)$ , $W_{f}$ is the number of real molecules represented by one sample molecule, $N_{\mathrm{tot}}\left(i,j\right)$ is the total number of molecules sampled at cell $\left(i,j\right)$ , $N^{\left(l\right)}\left(i,j\right)$ is the number of molecules sampled at the cell $\left(i,j\right)$ at the moment of l th sampling, and $c_{q}$ is the velocity of the q th sample molecule existing in the DSMC cell $\left(i,j\right)$ at the moment of the l th sampling. Note that the corresponding pressure is evaluated from $p_{i,j}=n_{i,j}k_{B}T_{i,j}$ . On the other hand, the propulsive force referred to in this study is the force acting tangentially on the bottom surface of the upper object, simply called in this study the tangential Knudsen force. The local tangential Knudsen stress, $\tau_{\mathrm{Kn},i}$ , i.e., the local tangential Knudsen force per unit area, is defined as

(7) $$\tau_{\mathrm{Kn},i}=\frac{W_{f}}{\Delta x_{i}\left(t_{\mathrm{end}}-t_{\mathrm{start}}\right)}\overset{N_{\mathrm{wall}-\mathrm{coll},i}}{\underset{q=1}{\sum }}m\left[{\left(c_{q}\right)}_{x}-{\left(c_{q}\right)}_{x}^{\prime }\right]$$

Here, $\tau_{\mathrm{Kn},i}$ is the tangential Knudsen stress exerted on the i th surface element with width $\Delta x_{i}$ on the bottom surface of the upper object, $t_{\mathrm{start}}$ and $t_{\mathrm{end}}$ are the starting time and the ending time of sampling, $N_{\mathrm{wall}-\mathrm{coll},i}$ is the number of sample molecules colliding onto the element $\Delta x_{i}$ during the sampling term from $t=t_{\mathrm{start}}$ to $t=t_{\mathrm{end}}$ , $c_{q}$ and $c_{q}^{\prime }$ are the pre-collision and post-collision velocities of the q th sample molecule in terms of the order of molecule–wall collision occurring in the sampling term, and the suffix x represents the x component of the molecular velocities. The net tangential Knudsen stress, $\tau_{\mathrm{Kn}}$ , is then obtained by averaging the local tangential Knudsen force for Equation (7).

## 3. Results and Discussion

**Figure 3:**
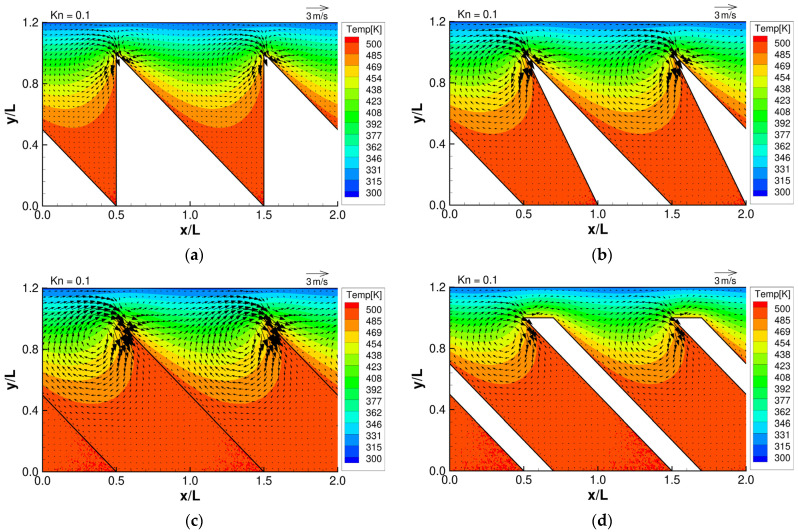
Flow distributions and temperature distributions for (**a**) ratchet, (**b**) modified ratchet, (**c**) oblique plate, and (**d**) oblique ridge, at Kn=0.1 .

**Figure 4:**
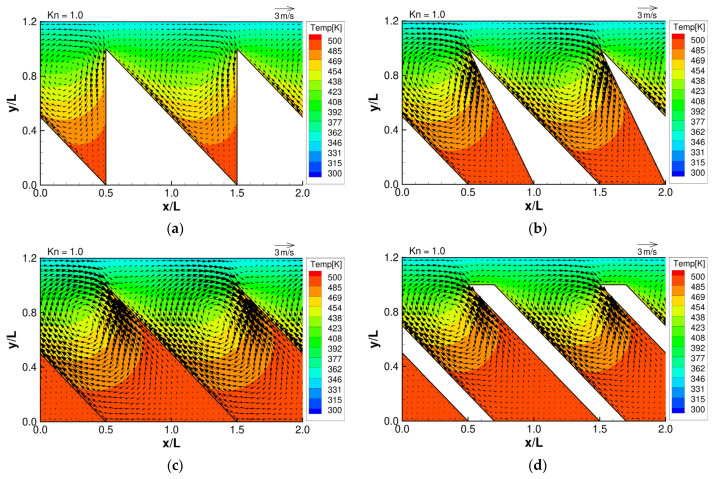
Flow distributions and temperature distributions for (**a**) ratchet, (**b**) modified ratchet, (**c**) oblique plate, and (**d**) oblique ridge, at Kn=1 .

**Figure 5:**
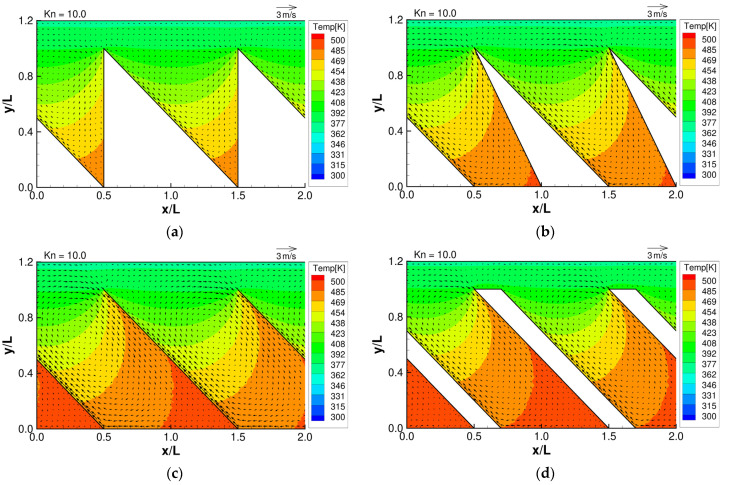
Flow distributions and temperature distributions for (**a**) ratchet, (**b**) modified ratchet, (**c**) oblique plate, and (**d**) oblique ridge, at Kn=10 .

From the works of Y. Sone and K. Aoki [1,2], with some knowledge of the expected temperature distribution in the gas, we can predict the behavior of the thermally induced gas flows mentioned in Section 1. Specifically, we expect that the temperature distribution of the gas near the oblique side will be non-uniform, and therefore, thermal stress slip flow can be induced near that region. In addition, we also expect that the temperature distribution at the tips of the microstructure will be strongly non-uniform, and therefore, strong thermal edge flows can be induced near that region. The strength and behavior of thermal edge flow will depend on the shape and orientation of the tips of the microstructure. As the tip angle is different in each microstructure, we expect that the strength and behavior of the thermal edge flow will vary in each configuration. Furthermore, according to Aoki et al. [2], the strength of thermally driven flows will depend on the Knudsen number. Figure 3, Figure 4 and Figure 5 show the resulting flow distributions and temperature distributions obtained from the DSMC simulation for each microstructure at selected Knudsen numbers. First, at Kn=0.1, it can be seen from Figure 3 that strong temperature gradients are localized on the tips of the microstructures, and strong flow is induced along the surfaces of the tips that are directed from the cold to the hotter region. This is the thermal edge flow that dominates the flow field, while the thermal stress slip flow is not visible. Second, at Kn=1 , it can be seen from Figure 4 that strong temperature gradients can now be seen in the vicinity of the oblique sides, and flows are induced along their surfaces from the hot region to the colder region. This is the thermal stress slip flow which combines with thermal edge flow to generate a strong counter-clockwise vortex that dominates the flow field. Lastly, at Kn=1 0, it can be seen from Figure 5 that the temperature gradients weaken, and the thermally driven flows also weaken but still persist, especially the thermal stress slip flow. As we expected, the sharper tips of the new configurations result in stronger thermal edge flows for all the selected Knudsen numbers. As for the flow along the oblique side, we can see stronger flow in the cases of the three new configurations compared to the ratchet configuration. However, the contribution of thermal stress slip flow, in this case, may not largely differ among the four configurations. This is because for all the configurations, the orientation of the oblique side is the same, and hence, the temperature field near the oblique side is not so different. Most probably, the difference in the flow along the oblique side among the cases is caused by the difference in the strength of the thermal edge flow to satisfy the equation of continuity. Thus, it was found that the three new configurations proposed in this paper induce much stronger flows compared to the conventional ratchet configuration. The results obtained here indicate that the new configurations can induce higher gas flow rates in the case of Knudsen pump applications.

**Figure 6:**
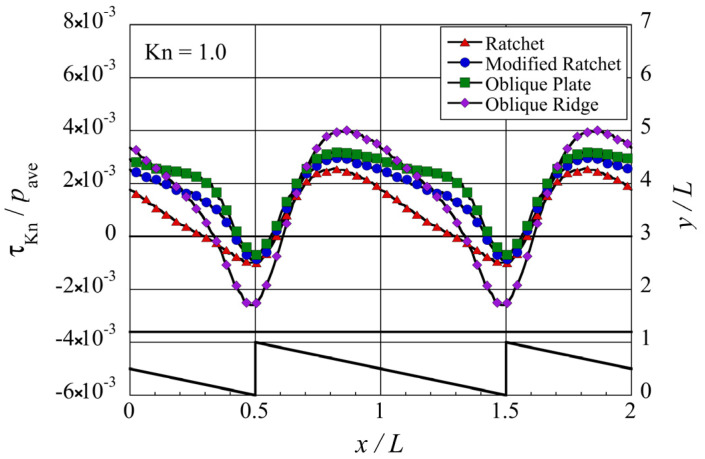
Distribution of the local tangential Knudsen stress, i.e., local propulsive force per unit area, for each case of the microstructure, at Kn=1 . The silhouette of the ratchet structure is added for easy reference.

Next, let us investigate the propulsive force induced on the bottom surface of the upper object. As mentioned, this propulsive force per unit area is the tangential Knudsen stress, $\;\tau_{\mathrm{Kn}}$ . Figure 6 shows the distribution of the local tangential Knudsen stress obtained from the DSMC simulation in each microstructure at Kn=1 . In this study, $p_{\mathrm{ave}}$ , which is the pressure spatially averaged in the bulk region of the gas, is used for non-dimensionalization. A positive value of the local tangential Knudsen stress means that the local propulsive force is directed rightward, i.e., +x -direction, while a negative value means that the local propulsive force is directed leftward, i.e., −x -direction. It can be seen that negative tangential Knudsen forces are located on the surface near the tip of each microstructure, while positive tangential Knudsen forces are located on the surface above the oblique side except in the region near the tip. When compared to other sections on the bottom surface of the upper object, the tangential Knudsen stress on the sections in the upper right neighborhood of the tip, i.e., around 0.5<x/L<0.7 , are similar in each configuration except for the case of the oblique ridge. Although the location of the tip for the oblique ridge is the same as that for the other configurations, the location of the oblique side is shifted rightward by its width w=0.2L . Therefore, care should be taken when comparing it with others. The differences in the distribution of the tangential Knudsen stress among the different configurations except for the oblique ridge can be considered to be largely due to the decrease in the tip angle β . 

**Figure 7:**
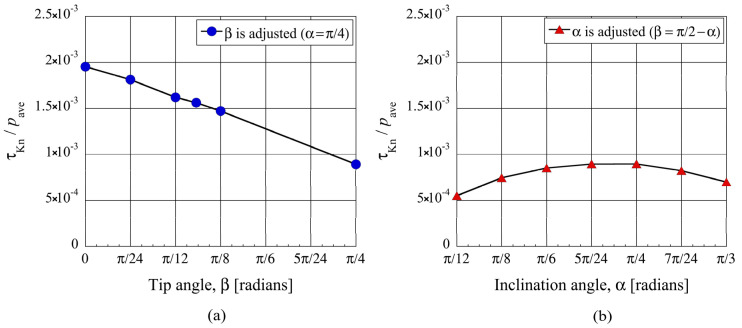
Net tangential Knudsen stresses, i.e., propulsive forces per unit area, at (**a**) different tip angles β for the modified ratchet and (**b**) different inclination angles α for the ratchet, for Kn=1 .

By integrating the distribution of local tangential Knudsen stress at Kn=1 , we obtain the net tangential Knudsen stress. Figure 7a shows the net tangential Knudsen stresses for different tip angle β under the condition of constant inclination angle α=π/4 . Here, the marker for β=π/4 represents the net tangential Knudsen stress for the ratchet, the marker for β=0 represents that for the oblique plate, and the other markers represent those for the modified ratchet. It can be seen that at constant inclination angle α=π/4 , the net tangential Knudsen stress increases linearly with decreasing tip angle β . Therefore, in addition to the fact that smaller tip angles result in stronger gas flows, as mentioned previously, the smaller tip angles also result in stronger propulsive force. Next, let us investigate the relationship between the net tangential Knudsen stress and the inclination angle α by adjusting the periodic length L while keeping the depth d of the ratchet structure. Figure 7b shows the resulting net tangential Knudsen stress for the ratchet for different inclination angle α , at Kn=1 . From the plot, the maximum tangential Knudsen stress is around α=π/4 , or when d=L , which is what we used in this study as a reference. Therefore, the propulsive force is maximum in the case that the aspect ratio of the microstructure is unity. As for the case α→π/2 , the geometry of the microstructure approaches that of a vertical plate structure which is symmetric in the horizontal direction, and the net tangential Knudsen stress, i.e., propulsive force, vanishes. While for the case α→0 , the substrate approaches that of a flat surface and the tangential Knudsen stress, i.e., propulsive force, also vanishes. The results obtained here on the relationship between the inclination angle α and the tangential Knudsen stress are similar to that obtained by Donkov et al. [16] even though we used fully diffusive walls, whereas they used a combination of specular and diffusive walls.

**Figure 8:**
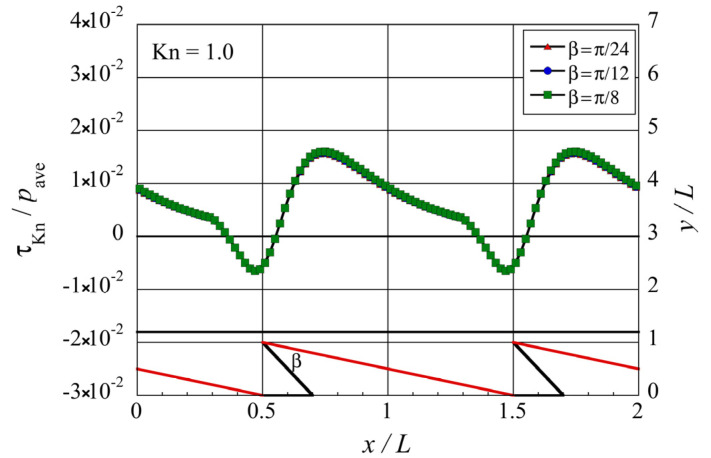
Distributions of the local tangential Knudsen stress due to molecules coming from the oblique side of the modified ratchet microstructure for different tip angles β at Kn=1 .

**Figure 9:**
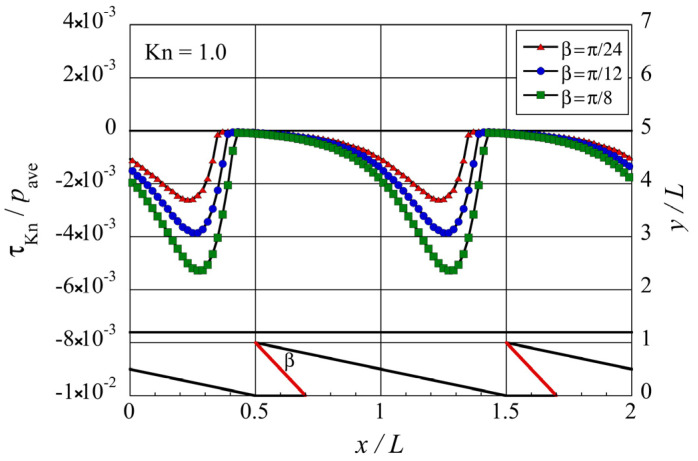
Distributions of the local tangential Knudsen stress due to molecules coming from the modified side of the modified ratchet microstructure, for different tip angles β at Kn=1 .

To understand how the tip angle β or how the shape of the structure, in general, affects the tangential Knudsen stress, i.e., propulsive force, let us consider two groups of molecules. First, those molecules diffusively reflected at the oblique side of the structure after impinging on it. Second, those molecules diffusively reflected at the modified side of the structure, i.e., the vertical side in the case of ratchet, after impinging on it. Note that both groups of molecules obtain higher momentum compared with molecules in bulk during the process of diffuse reflection due to the high temperature of the walls. Due to the orientation of the walls, the former group of molecules carries a +x -momentum, while the latter group of molecules carries a −x -momentum. When these two groups of molecules reach the bottom surface of the upper object without experiencing an intermolecular collision in the gas, they transfer their x -momentum onto the bottom surface of the upper object. In the case where all the molecules arriving at the bottom surface of the upper object come from the gas after experiencing intermolecular collisions there, most probably, the x-momentum brought on it is not so large. However, if some of the impinging molecules are replaced by the molecules with higher momentum directly coming from the hot sides of the microstructure, the imbalance in the x-momentum flux will be induced by such molecules, and hence, a net propulsive force will occur. The imbalance occurs based on two factors: distance from the microstructure to the bottom surface of the upper object and orientation of the modified side. When molecules travel a longer distance, they have more chance to collide with other molecules and cannot bring the x -momentum onto the bottom surface of the upper object. As the tip angle β is decreased, the distance between the surface element on the bottom surface of the upper object and the modified side increases, and hence the x -momentum carried by reflected molecules leaving the modified side also decreases. Furthermore, when the orientation of the modified side deviates from a vertical position such that it is rotated counter-clockwise, the number flux of reflected molecules towards the bottom surface of the upper object decreases. The combination of these factors means that decreasing the tip angle β decreases the negative contribution of the second group of molecules, i.e., those that come from the modified side to the propulsive force. On the other hand, since we kept the orientation of the oblique side, i.e., α is constant, the contribution of the first group of molecules, i.e., those that come from the oblique side to the propulsive force, is unchanged. As only the negative contribution of the second group of molecules which come from the modified side is decreased, the resultant rightward force increases with decreasing tip angle β . Figure 8 shows the distribution of the local tangential Knudsen stress due to the first group of molecules, i.e., those coming from the oblique side of the structure, and Figure 9 shows the distribution of the local tangential Knudsen stress due to the second group of molecules, i.e., those coming from the modified side of the structure. Both figures show the results for the modified ratchet. Figure 8 verifies our discussion above that the contribution of the first group of molecules remains unchanged at different tip angle β . However, Figure 9 verifies our discussion above that the negative contribution of the second group of molecules decreases at decreasing tip angle β .

**Figure 10:**
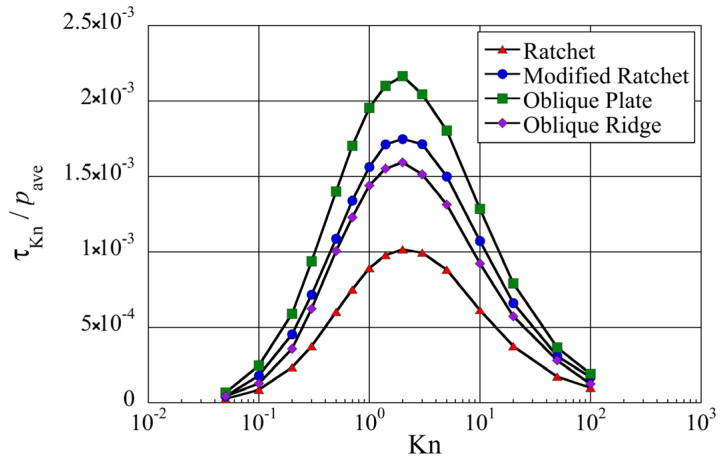
Net tangential Knudsen stresses, i.e., propulsive forces per unit area, at different Knudsen numbers for different surface microstructures.

Let us look at how the propulsive force is affected by the Knudsen number. By integrating the distribution of local tangential Knudsen stress, i.e., propulsive force per unit area, the net tangential Knudsen stress is obtained at different Knudsen numbers, as shown in Figure 10. It was found that a net positive tangential Knudsen stress is obtained, i.e., the propulsive force induced on the object is directed rightward or in the +x -direction, for all Knudsen numbers irrespective of the configuration of the microstructure. However, we can clearly see that the magnitude of the net tangential Knudsen stress varies depending on the structure. In fact, the oblique plate induces a net tangential Knudsen stress that is about two times larger than that of the ratchet. If we rank the configurations in terms of the strongest propulsive force, the oblique plate is followed by a modified ratchet and oblique ridge, which induce about 1.5 times larger propulsive force than that of the ratchet.

**Figure 11:**
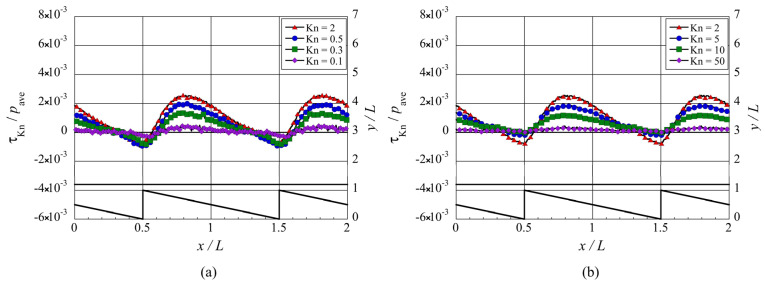
Distributions of the local tangential Knudsen stress, i.e., local propulsive force per unit area, for the ratchet microstructure, at (**a**) selected lower Knudsen numbers, Kn≤2 , and (**b**) selected higher Knudsen numbers, Kn≥2 .

Although the magnitude of the net tangential Knudsen stress varies significantly with the type of configuration, their distribution follows a similar trend with the Knudsen number. The net propulsive force vanishes at low Knudsen numbers, Kn<0.1 , i.e., near the continuum flow limit, peaks at Kn=2 , i.e., in the transition flow regime, and vanishes at very high Knudsen numbers Kn>100 , i.e., near the free molecular flow limit. To support this, consider Figure 11 which shows the distribution of the local tangential Knudsen stress in the case of the ratchet microstructure. It can be seen in Figure 11a that at lower Knudsen numbers, i.e., Kn≤2 , the local tangential Knudsen stress weakens with decreasing Knudsen number. This is consistent with the fact that in the continuum flow regime, i.e., Kn→0 , the gas is in equilibrium condition, and hence, the thermally induced flows and tangential Knudsen stress vanish. Furthermore, Figure 11b shows that at higher Knudsen numbers, i.e., Kn≥2 , the local tangential Knudsen stress weakens with increasing Knudsen number. This agrees with the work of Donkov et al. [16], where it is mentioned that in the case of a channel bounded by fully diffusive surfaces, the tangential Knudsen stress vanishes in the free-molecular flow regime, i.e., Kn→∞ . Although the distributions in Figure 11 are for the ratchet microstructure, the same trend can be seen in the cases of the other microstructures presented in this study.

**Figure 12:**
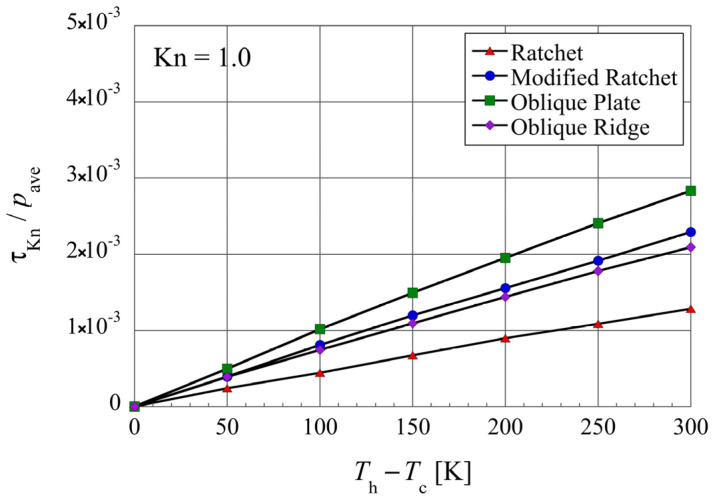
Net tangential Knudsen stresses, i.e., propulsive forces per unit area, for different surface microstructures at different temperature differences, in the case of Kn=1 and $\left(T_{h}+T_{c}\right)/2=400\;K$ .

Lastly, let us look at the effect of the temperature difference between the heated microstructure and the colder upper object, i.e., $T_{h}-T_{c}$ , on the net propulsive force. Figure 12 shows the distribution of the net propulsive force in each of the microstructures at various temperature differences, at Kn=1 , where the temperature difference $T_{h}-T_{c}$ is changed while the middle value $\left(T_{h}+T_{c}\right)/2$ between the surface temperatures $T_{c}$ and $T_{h}$ is kept at 400 K . It can be seen that in the case of no temperature difference, i.e., $T_{h}=T_{c}$ , the net tangential Knudsen stress vanishes in all the microstructures. As the temperature difference is increased, the net tangential Knudsen stress increases linearly, whose slope varies depending on the microstructure. Here, the oblique plate has the largest slope, followed by the modified ratchet and the oblique ridge, while the conventional ratchet has the smallest slope.

In this study, we have shown that the self-propulsion phenomenon is optimized in the case of the oblique plate configuration. From the perspective of manufacturing, a structure with no thickness is difficult, and so an oblique ridge is an alternative to the oblique plate, where the induced propulsive force is still stronger than the conventional ratchet microstructure.

## 4. Conclusions

In this study, we considered four surface microstructures, one of which is the conventional ratchet configuration, and the three configurations are modifications of the ratchet. Using the DSMC method, we have demonstrated that these new configurations can induce stronger thermally driven gas flows and a larger propulsive force on the object placed close to them. The differences in the induced flows among these microstructures are due to the fact that these new configurations have smaller tip angles than the ratchet configuration, which results in stronger thermal edge flows. Similarly, the differences in the propulsive force among these microstructures are also attributed to their differences in the tip angle. That is, for smaller tip angles, the negative contribution to the rightward propulsive force due to molecules impinging on the bottom surface of the upper object directly from the modified side without experiencing intermolecular collision is reduced, and as a result, the rightward propulsive force increases with decreasing tip angle. In addition to the fact that the magnitude of the propulsive force depends on the microstructure, the Knudsen number and the temperature difference also significantly affect the propulsive force. For all the microstructures considered in this study, it was found that the propulsive force vanishes near the continuum flow regime and near the free molecular flow regime and is maximum in the transition flow regime. Furthermore, it was also found that for all the microstructures considered here, the propulsive force increases linearly with the increasing temperature difference between the heated microstructure and the colder object. The results show that although the oblique plate microstructure induces the strongest propulsive force, both the modified ratchet and oblique ridge microstructures still induce a stronger propulsive force compared to the conventional ratchet microstructure. 

## Figures and Tables

**Table 1:** Parameters for the DSMC Simulation for the different Knudsen number cases.

Knudsen Number	Cell Size	Time Step
0.05, 0.1	$\lambda_{\mathrm{ref}}/3$	$0.08\tau_{\mathrm{ref}}$
0.2, 0.3, 0.5, 0.7, 1.0	0.05g	$0.08\tau_{\mathrm{ref}}$
1.4, 2.0, 3.0, 5.0, 10, 20, 50, 100	0.05g	$0.08g/{\bar{C}}_{\mathrm{ref}}$

## Data Availability

Data sources can be provided upon request.

## References

[1] Sone Y. (2007). Molecular Gas Dynamics: Theory, Techniques, and Applications.

[2] Aoki K., Takata S., Aikawa H. (2001). A rarefied gas flow caused by a discontinuous wall temperature. Phys. Fluids.

[3] Wang X., Su T., Zhang W., Zhang Z., Zhang S. (2020). Knudsen pumps: A review. Microsyst. Nanoeng..

[4] Ebrahimi A., Roohi E. (2017). DSMC investigation of rarefied gas flow through diverging micro- and nanochannels. Microfluid Nanofluid.

[5] Zhang Z., Wang X., Zhao L., Zhang S., Zhao F. (2019). Study of Flow Characteristics of Gas Mixtures in a Rectangular Knudsen Pump. Micromachines.

[6] Hssikou M., Baliti J., Alaoui M. (2018). Numerical analysis of non-isothermal walls driven-gas flow. Int. J. Eng. Syst. Model. Simul..

[7] Hssikou M., Baliti J., Alaoui M. (2019). Continuum Analysis of Rarefaction Effects on a Thermally Induced Gas Flow. Math. Probl. Eng..

[8] Lotfian A., Roohi E. (2019). Radiometric flow in periodically patterned channels: Fluid physics and improved configurations. J. Fluid Mech..

[9] Baier T., Hardt S., Shahabi V., Roohi E. (2017). Knudsen pump inspired by Crookes radiometer with a specular wall. Phys. Rev. Fluids.

[10] Wu Y. (1967). Kinetic Theory of Molecular Radiometric Force and Radiometer. Ann. Phys..

[11] Yamamoto K., Ishihara Y., Fujise K. (1988). Thermophoresis of a Circular Cylinder in a Rarefied Gas. J. Phys. Soc. Jpn..

[12] Lereu A.L., Passian A., Warmack R.J., Ferrell T.L., Thundat T. (2004). Effect of thermal variations on the Knudsen forces in the transitional regime. Appl. Phys. Lett..

[13] Sugiura Y. (1954). Experimental Studies on the Force exerted on a Disc placed in a Flow of Rarefied Gas. J. Phys. Soc. Jpn..

[14] Strongrich A., Alexeenko A. (2015). Microstructure actuation and gas sensing by the Knudsen thermal force. Appl. Phys. Lett..

[15] Passian A., Wig A., Meriaudeau F., Ferrel T.L., Thundat T. (2002). Knudsen forces on microcantilevers. J. Appl. Phys..

[16] Donkov A., Tiwari S., Liang S., Hardt S., Klar A., Ye W. (2011). Momentum and mass fluxes in a gas confined between periodically structured surfaces at different temperatures. Phys. Rev. E.

[17] Hardt S., Tiwari S., Baier T. (2013). Thermally driven flows between a Leidenfrost solid and a ratchet surface. Phys. Rev. E.

[18] Shahabi V., Baier T., Roohi E., Hardt S. (2017). Thermally induced gas flows in ratchet channels with diffuse and specular boundaries. Sci. Rep..

[19] Würger A. (2011). Leidenfrost Gas Ratchets Driven by Thermal Creep. Phys. Rev. Lett..

[20] Bird G.A. (1994). Molecular Gas Dynamics and the Direct Simulation of Gas Flows.

[21] Nanbu K. (1980). Direct simulation scheme derived from the Boltzmann equation I. Monocomponent gases. J. Phys. Soc. Jpn..

[22] Vincenti W., Kruger C. (1965). Introduction to Physical Gas Dynamics.

[23] National Astronomical Observatory of Japan (2006). Rika Nenpyo (Chronological Scientific Tables 2007).

[24] Kaye G., Laby T. (1986). Tables of Physical and Chemical Constants.

[25] Nanbu K. (1992). Stochastic solution method of the Boltzmann equation I. Mem. Inst. Fluid. Sci..

[26] Nanbu K. (2000). Probability theory of electron–molecule, ion–molecule, molecule–molecule, and Coulomb collisions for particle modeling of materials processing plasmas and cases. IEEE T. Plasma Sci..

