# Correction: Otic, C.J.C.; Yonemura, S. Effect of Different Surface Microstructures in the Thermally Induced Self-Propulsion Phenomenon. *Micromachines* 2022, *13*, 871

**DOI:** 10.3390/mi13081181

**Published:** 2022-07-27

**Authors:** Clint John Cortes Otic, Shigeru Yonemura

**Affiliations:** 1 Department of Finemechanics, Graduate School of Engineering, Tohoku University, 6-6 Aramaki Aza Aoba, Aoba-ku, Sendai 980-8579, Miyagi, Japan; 2 Department of Mechanical Engineering, College of Engineering, Chubu University, 1200 Matsumoto-cho, Kasugai 487-8501, Aichi, Japan

The authors wish to make the following corrections to the published paper [1]. Figures 3–5, 7 and 11 were published in the incorrect format, in which the results were not displayed properly. In the corrected version, the authors have modified the figures from EPS format to high-resolution JPEG format. For consistency, all figures, i.e., Figure 1, Figure 2, Figure 3, Figure 4, Figure 5, Figure 6, Figure 7, Figure 8, Figure 9, Figure 10, Figure 11 and Figure 12, have been replaced in high-resolution JPEG format as appears in the succeeding pages.

The authors state that the scientific conclusions are unaffected. This correction was approved by the Academic Editor. The original publication has also been updated.

## Figures and Tables

**Figure 1:**
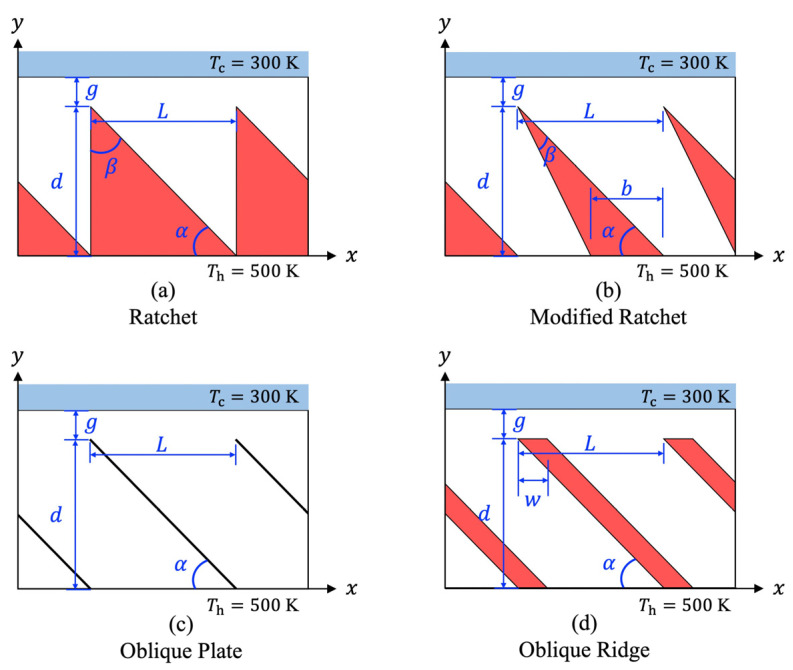
Schematics of the substrate with different surface microstructures.

**Figure 2:**
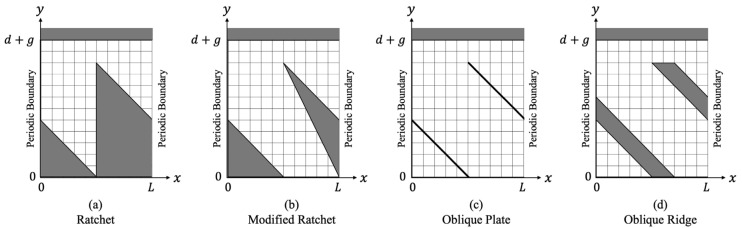
Computational domain used in each microstructure.

**Figure 3:**
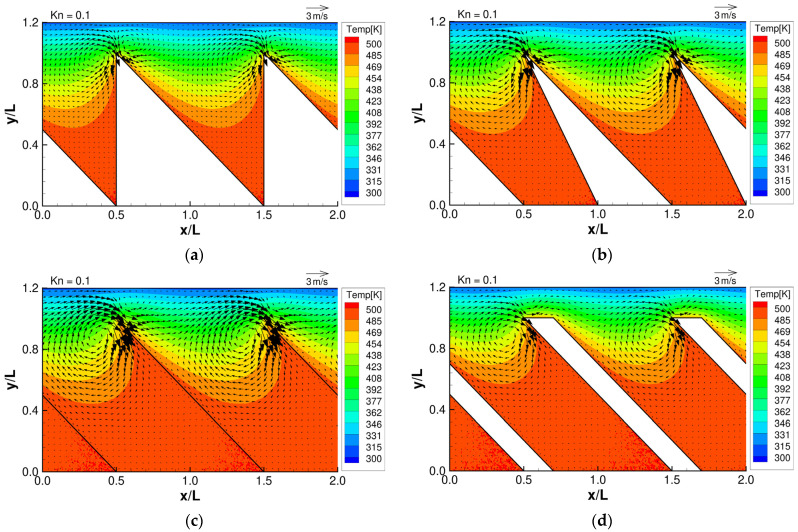
Flow distributions and temperature distributions for (**a**) ratchet, (**b**) modified ratchet, (**c**) oblique plate, and (**d**) oblique ridge, at Kn =0.1.

**Figure 4:**
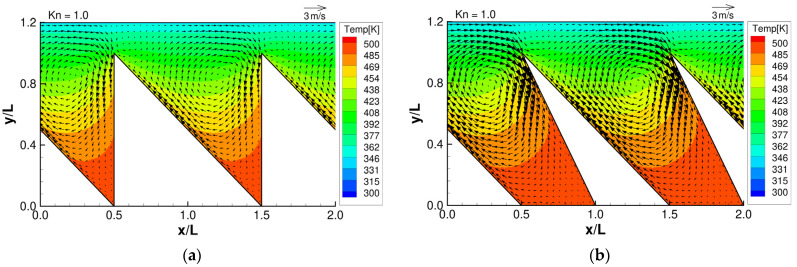

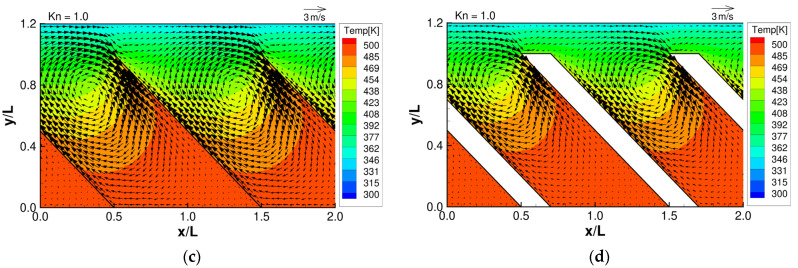
Flow distributions and temperature distributions for (**a**) ratchet, (**b**) modified ratchet, (**c**) oblique plate, and (**d**) oblique ridge, at Kn =1.

**Figure 5:**
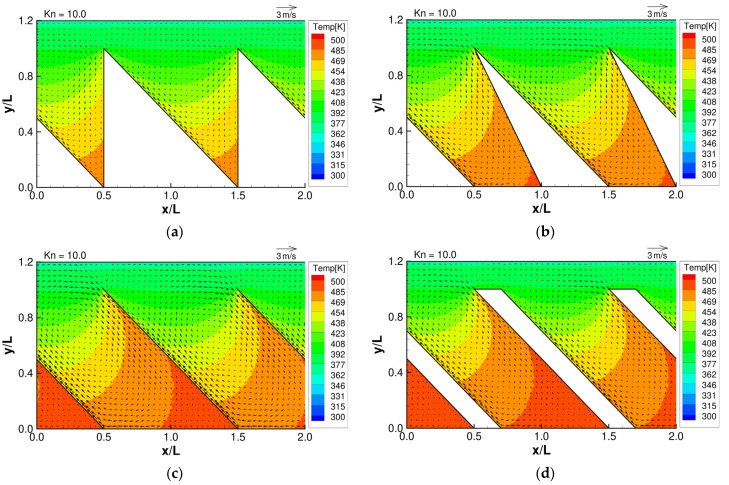
Flow distributions and temperature distributions for (**a**) ratchet, (**b**) modified ratchet, (**c**) oblique plate, and (**d**) oblique ridge, at Kn =10.

**Figure 6:**
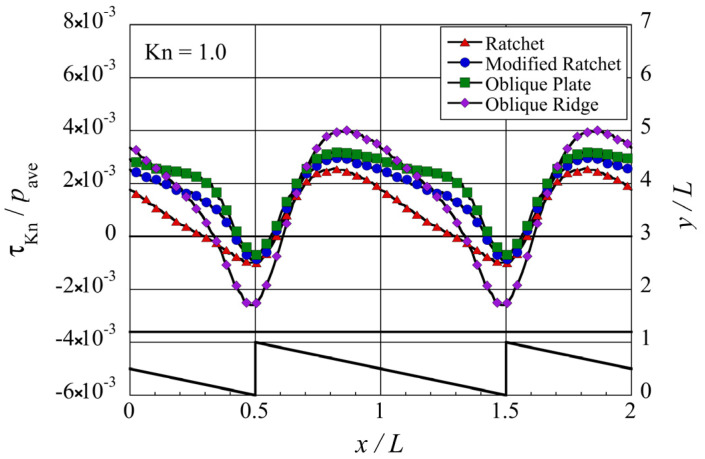
Distribution of the local tangential Knudsen stress, i.e., local propulsive force per unit area, for each case of the microstructure, at Kn=1. The silhouette of the ratchet structure is added for easy reference.

**Figure 7:**
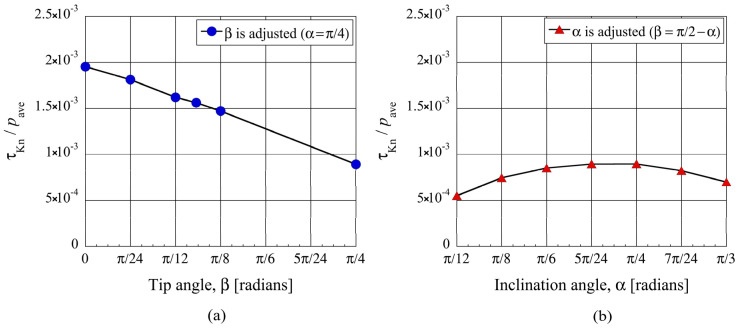
Net tangential Knudsen stresses, i.e., propulsive forces per unit area, at (**a**) different tip angles β for the modified ratchet and (**b**) different inclination angles α for the ratchet, for Kn=1.

**Figure 8:**
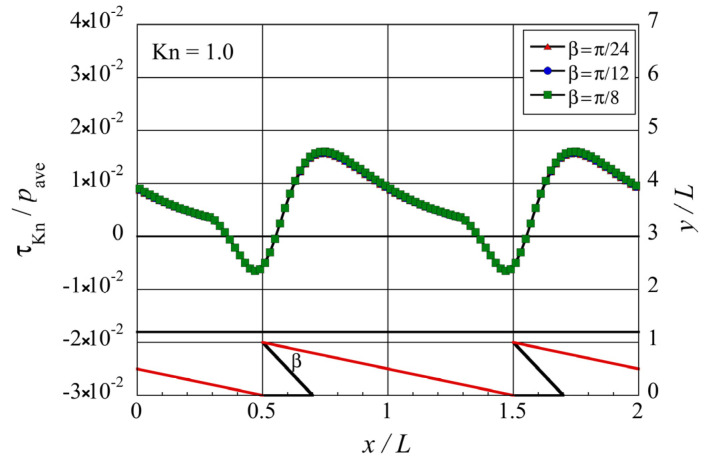
Distributions of the local tangential Knudsen stress due to molecules coming from the oblique side of the modified ratchet microstructure for different tip angles β at Kn=1.

**Figure 9:**
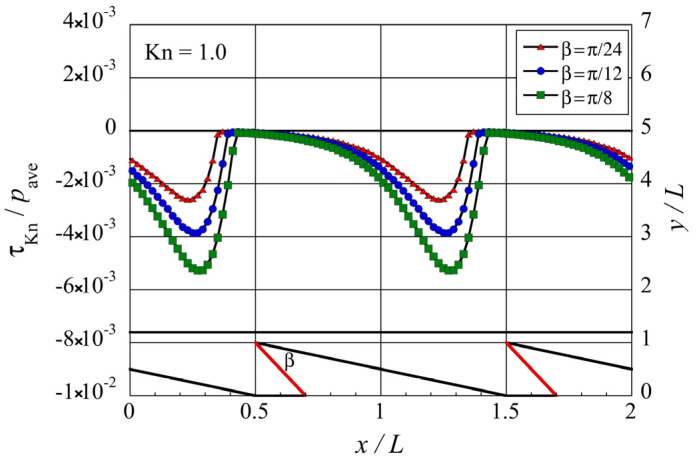
Distributions of the local tangential Knudsen stress due to molecules coming from the modified side of the modified ratchet microstructure, for different tip angles β at Kn=1.

**Figure 10:**
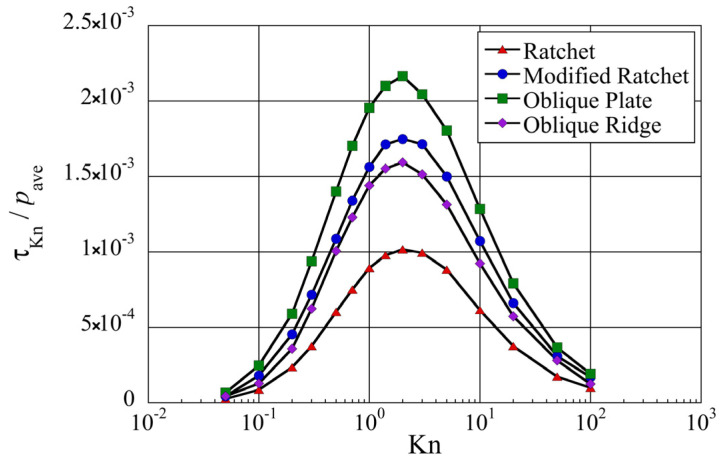
Net tangential Knudsen stresses, i.e., propulsive forces per unit area, at different Knudsen numbers for different surface microstructures.

**Figure 11:**
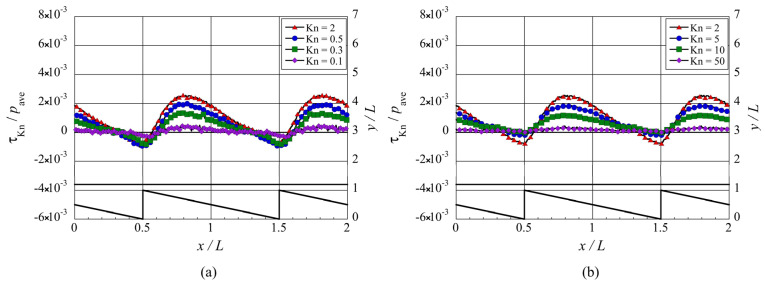
Distributions of the local tangential Knudsen stress, i.e., local propulsive force per unit area, for the ratchet microstructure, at (**a**) selected lower Knudsen numbers, Kn≤2, and (**b**) selected higher Knudsen numbers, Kn≥2.

**Figure 12:**
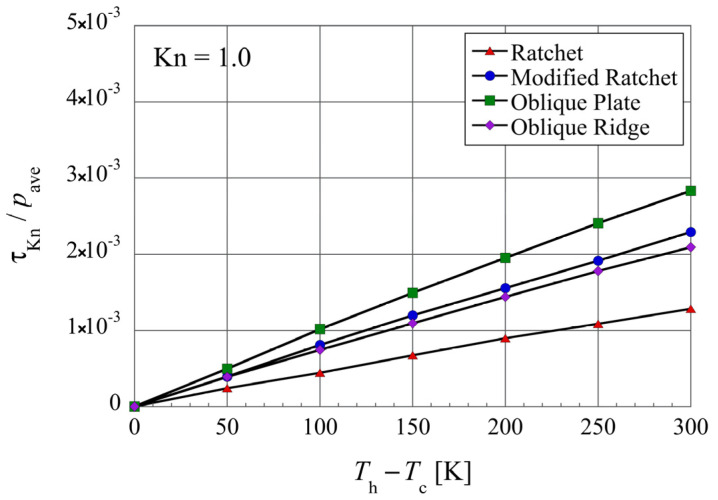
Net tangential Knudsen stresses, i.e., propulsive forces per unit area, for different surface microstructures at different temperature differences, in the case of Kn=1 and $\left(T_{h}+T_{c}\right)/2=400\;K$.
